# Plasmid Copy Number and Disease Severity in Naturally Occurring Ocular Chlamydia trachomatis Infection

**DOI:** 10.1128/JCM.02618-13

**Published:** 2014-01

**Authors:** Anna R. Last, Chrissy h. Roberts, Eunice Cassama, Meno Nabicassa, Sandra Molina-Gonzalez, Sarah E. Burr, David C. W. Mabey, Robin L. Bailey, Martin J. Holland

**Affiliations:** a Clinical Research Department, London School of Hygiene and Tropical Medicine, London, United Kingdom; b Programa Nacional de Saúde de Visão, Ministério de Saúde Publica, Bissau, Guinea Bissau; c Disease Control and Elimination Theme, Medical Research Council Unit The Gambia, Fajara, The Gambia

## Abstract

The Chlamydia trachomatis plasmid is a virulence factor. Plasmid copy number, C. trachomatis load and disease severity were assessed in a treatment-naive population where trachoma is hyperendemic. By using droplet digital PCR, plasmid copy number was found to be stable (median, 5.34 [range, 1 to 18]) and there were no associations with C. trachomatis load or disease severity.

## TEXT

Trachoma is caused by infection with ocular strains of Chlamydia trachomatis. The 7.5-kb C. trachomatis plasmid has been shown to function as a virulence factor in animal models (1, 2). Phenotypic differences exist between plasmid-cured and wild-type C. trachomatis strains with respect to infectivity, glycogen accumulation, induction of inflammation, and activation of Toll-like-receptor pathways (3, 4). Plasmid deletion mutagenesis studies showed that deletion of the plasmid-borne *pgp4* gene results in an *in vitro* phenotype identical to that of a plasmid-free strain (5). This supports bacterial transcriptome analysis showing a decrease in transcript levels of a subset of chromosomal genes in a naturally occurring plasmid-free strain of C. trachomatis, demonstrating that the plasmid is a transcriptional regulator of virulence-associated genes (6).

There is little information in the literature relating plasmid copy number (per genome) to virulence (7–9). The mechanisms of plasmid virulence are not clearly defined, particularly in naturally occurring infections. We assessed plasmid copy number variation and its association with disease severity in ocular C. trachomatis infection from a treatment-naive population on the Bijagós Archipelago of Guinea Bissau where trachoma is hyperendemic.

This study was conducted in accordance with the declaration of Helsinki. Ethical approval was obtained from the Comitê Nacional de Ética e Saúde (Guinea Bissau), the LSHTM Ethics Committee (United Kingdom), and the Gambia Government/MRC Joint Ethics Committee (The Gambia). Written (thumbprint or signature) informed consent was obtained from all study participants or their guardians as appropriate. Following the survey all communities on the study islands were treated with azithromycin in line with WHO and national protocols.

Individuals from 300 randomly selected households from 38 villages on four islands were examined by a single trained examiner using the simplified WHO and modified FPC grading systems (10, 11). In the FPC system, follicles (F), papillae (P), and conjunctival scarring (C) are separately scored on a scale of 0 to 3. Active disease (TF [follicular trachoma] or TI [inflammatory trachoma] according to the simplified WHO system) equates to F2/3 and P3, respectively. C2/3 (and in some cases C1) is equivalent to TS (trachomatous scarring). Both systems were used to provide detailed phenotypic information and comparability with other studies. Individuals' age, sex, and ethnicity were recorded.

Swabs were taken from the left upper tarsal conjunctiva of each participant using a validated procedure (12, 13). Swabs were collected dry into microcentrifuge tubes (Simport, Canada), kept on ice in the field, and frozen to −80°C within 8 h of collection. Measures were taken to avoid cross-contamination in the field and in the laboratory (13).

DNA extraction and droplet digital PCR (ddPCR) for detection of C. trachomatis plasmid were conducted as described previously (14). A second duplex assay was used to estimate plasmid and chromosome (*omcB*) target concentrations within the same reaction in plasmid-positive samples. We used published primer-probe target sequences appropriate for quantitation of all genovars of C. trachomatis (7, 14). We used a modified *omcB* probe to improve quenching efficiency and reduce background fluorescence (Table 1). Methods for master mix preparation, droplet generation, thermal cycling conditions, droplet reading, target DNA concentration calculation, and retesting of saturated samples are described elsewhere (14). Estimated quantities of *omcB* and plasmid are expressed as copies/swab. C. trachomatis load refers to *omcB* copies/swab. Plasmid copy number (per genome) was calculated using the plasmid/genome ratio.

**TABLE 1:** Primer and probe sequences for control and C. trachomatis targets using the ddPCR system^*a*^

Molecular target and primer or probe	Nucleotide sequence and modifications
Homo sapiens RNase P/MRP 30-kDa subunit (RPP30) (internal control)	
Forward primer (RPP30-F)	5′ AGA TTT GGA CCT GCG AGC G 3′
Reverse primer (RPP30-R)	5′ GAG CGG CTG TCT CCA CAA GT 3′
Probe (RPP30_HEX_BHQ1)	5′ HEX-TTC TGA CCT GAA GGC TCT GCG CG-BHQ1 3′
C. trachomatis cryptic plasmid pLGV440 (circular; genomic DNA; 7,500 bp)	
Forward primer (Ct-plasmid-F)	5′ CAG CTT GTA GTC CTG CTT GAG AGA 3′
Reverse primer (Ct-plasmid-R)	5′ CAA GAG TAC ATC GTT CAA CGA AGA 3′
Probe (Ct-plasmid_FAM_BHQ1)^*b*^	5′ 6FAM-CCC CAC CAT TTT TCC GGA GCG A-BHQ1 3′
Probe (Ct-plasmid_HEX_BHQ1)^*c*^	5′ HEX-CCC CAC CAT TTT TCC GGA GCG A-BHQ1 3′
C. trachomatis (serovar A) *omcB* gene	
Forward primer (Ct-*omcB*-F)	5′ GAC ACC AAA CGC AAA GAC AAC AC 3′
Reverse primer (Ct-*omcB*-R)	5′ ACT CAT GAA CCG GAG CAA CCT 3′
Probe (Ct-*omcB*-FAM-BHQ1)	5′ 6FAM-CCA CAG CAA AGA GAC TCC CGT AGA CCG-BHQ1 3′

a MRP, mitochondrial RNA processing endoribonuclease; 6FAM, 6-carboxyfluorescein reporter; BHQ1, black hole quencher 1; HEX, hexachlorofluorescein reporter.

b C. trachomatis plasmid probe used in screening (first) assay.

c C. trachomatis probe used in quantitative (second) assay.

Raw quantitation data were processed as previously described (14). Geometric mean *omcB* load and linear and logistic regression analyses (with odds ratios [OR]) were conducted in STATA 12 (Stata Corporation, College Station, TX) to examine associations between plasmid copy number, load, and detailed clinical phenotype. C. trachomatis load and plasmid copy number data were log(e) transformed, and robust standard error was used where indicated.

Of 1,511 individuals enrolled, 1,508 individuals consented to ocular assessment, and 1,507 conjunctival swabs were obtained. The median age of participants was 13 years (1 month to 88 years), and 57% were female. Most participants were of the Bijagós ethnic group. The prevalence of active trachoma (TF/TI) in 1- to 9-year-olds was 21% (136/660) (95% confidence interval [CI], 17.89 to 24.11%). Overall, 11% had clinically active trachoma (164/1,508) (95% CI, 9.42 to 12.58%). C. trachomatis plasmid DNA was detected in 16% overall (233/1,507) (26% of 1- to 9-year-olds). All samples were adequate according to criteria described previously (14).

C. trachomatis load was estimated in 79% (184/233) of plasmid-positive samples. In 21% of samples where plasmid load was very low, *omcB* was below the level of detection.

The geometric mean estimated number of *omcB* copies/swab varied by clinical phenotype: 294 copies/swab (95% CI, 165 to 524) in 73 subjects with normal conjunctivae, 8,562 copies/swab (95% CI, 5,412 to 13,546) in 92 with active trachoma, and 928 copies/swab (95% CI, 280 to 2,074) in 19 with scarring.

**FIG 1:**
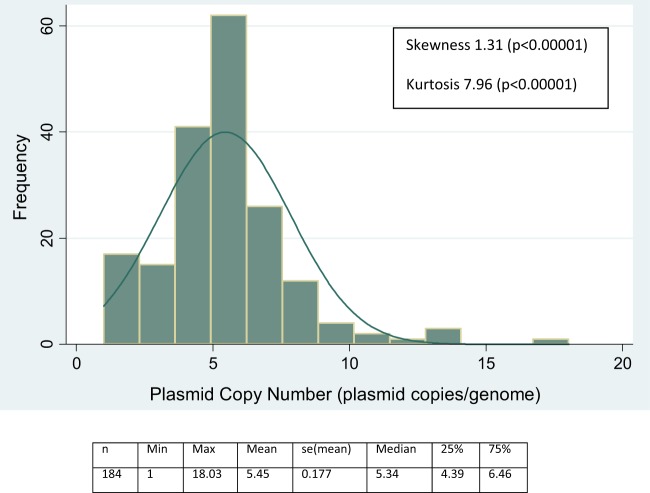
Distribution of plasmid copy number variation in naturally occurring ocular infections with C. trachomatis within the study population. se, standard error.

**FIG 2:**
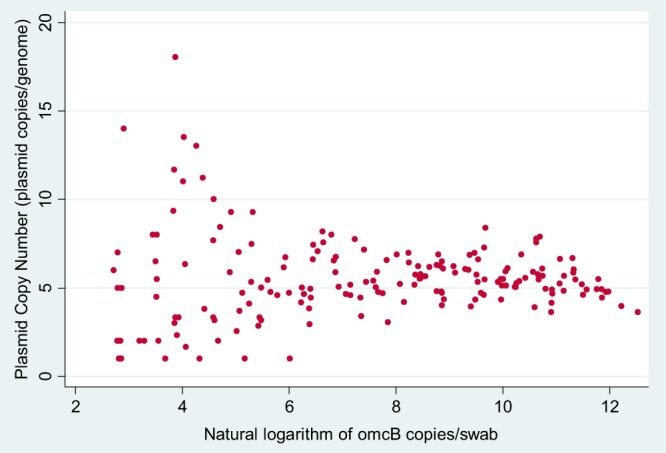
C. trachomatis load and plasmid copy number variation.

The median plasmid copy number was 5.34 (1 to 18.03) (Fig. 1). Plasmid copy number was stable in infections across the four study islands (Kruskal-Wallis H [χ^2^] = 4.5001 [df = 3; *P* = 0.2123]). Plasmid copy number was not associated with the presence of active trachoma (OR, 1.00; 95% CI, 0.88 to 1.12; *P* = 0.960), severity of inflammatory (OR, 1.04; 95% CI, 0.927 to 1.162; *P* = 0.515) or follicular (OR, 1.03; 95% CI, 0.922 to 1.159; *P* = 0.572) disease, or C. trachomatis load (Table 2). At lower loads, the variance was highly heterogeneous (Levene's *W*_0_ = 55.3; df = 2; *P* < 0.000000001) (Fig. 2).

**TABLE 2:** Relationship between plasmid copy number and C. trachomatis load^*a*^

No. of *omcB* copies/swab	No. of samples	Plasmid copy no.			
		Variance	Minimum	Median	Maximum
<100	41	19.8139	1	4.1514	18.0291
100–10,000	82	2.7136	1	5.3421	9.2819
>10,000	62	1.0814	3.6164	5.4261	8.3947

a Kruskall Wallis H (χ^2^) = 4.58; df = 2; *P* = 0.10.

The theoretical advantages of ddPCR are presented by Hindson et al. (15). These include nanoliter-sized droplet partitioning of the reaction, which promotes optimal primer-template interaction conditions robust to variation in PCR efficiency, thus enabling accurate estimation of both plasmid and *omcB* copy numbers within the same reaction. We have discussed the precision and accuracy of our diagnostic ddPCR assay elsewhere (14).

There are a few published studies examining plasmid copy number in reference strains of C. trachomatis (7–9, 16, 17). Pickett et al. showed that across 12 C. trachomatis serovars, the plasmid copy number was not significantly different, but there were variations depending on growth phase and condition during *in vitro* culture (7). Seth-Smith et al. showed an increased plasmid copy number in ocular relative to urogenital strains (8). We demonstrate a stable plasmid copy number distribution in naturally occurring ocular C. trachomatis infection that does not vary with geographic location, clinical phenotype, or C. trachomatis load. Our data show that ddPCR may have limitations in measuring plasmid copy number in very-low-load infections (<200 *omcB* copies/swab), where plasmid copy number variance is greatest. This observation may reflect a breakdown in the assumptions required to apply the Poisson distribution to accurately estimate load with ddPCR. Despite the caveats, our data suggest plasmid copy number stability in naturally occurring ocular C. trachomatis infection.

Maintenance of the plasmid at low copy numbers carries an inherent risk during cell partition (18), but naturally occurring plasmid-free strains are rare (19–21). A lower-risk, higher-copy-number system is metabolically expensive but may confer a fitness advantage. Thus, the maintenance of 5 or 6 plasmids per genome may maximize infectivity or intracellular survival while provoking minimal host immune response.

Though there is convincing evidence that the chlamydial plasmid is a virulence factor (3, 4, 6, 22–24), our data suggest that plasmid copy number is not associated with disease severity and that additive gene dosage effects do not appear to correlate with pathogen virulence *in vivo*. This supports *in vitro* work showing no association between plasmid copy number and tissue tropism (9). Previous work *in vitro* and in animal models suggests that subtle genomic differences between chlamydial isolates are associated with differences in growth kinetics, immune responses, and pathology (25, 26). Further epidemiological and *in vitro* studies using comparative pathogen genomics to examine these associations are required to fully understand the relationship between disease severity and chlamydial virulence.
